# Retraction Note: Chemokine receptor 7 overexpression promotes mesenchymal stem cell migration and proliferation via secreting Chemokine ligand 12

**DOI:** 10.1038/s41598-022-08386-8

**Published:** 2022-03-15

**Authors:** Ling Liu, Jian-Xiao Chen, Xi-Wen Zhang, Qin Sun, Lan Yang, Airan Liu, Shuling Hu, Fengmei Guo, Songqiao Liu, Yingzi Huang, Yi Yang, Hai-Bo Qiu

**Affiliations:** Department of Critical Care Medicine, School of Medicine, Nanjing Zhongda Hospital, Southeast University, Nanjing, 210009 China

Retraction of: *Scientific Reports* 10.1038/s41598-017-18509-1, published online 09 January 2018

The Editors have retracted this Article.

After publication, concerns were raised with the data presented in the Figures, specifically:

- In Figure 1a, flow cytometry graphs for MSC-OENC-CXCR7 and MSC-ShNC-CXCR7 appear to be identical, but have different % values.
- In Figure 4a, the two MSC-BLANK images are duplicated in the two experiments.
- In Figure 4b, the two MSC-BLANK images are also duplicated with different brightness levels.

The Authors did not provide the original data upon the Editors' request. The Editors therefore no longer have confidence in the presented data.

Ling Liu, Jian-Xiao Chen, Qin Sun, Lan Yang, Airan Liu, Shuling Hu, Fengmei Guo, Songqiao Liu, Yingzi Huang, Yi Yang and Hai-Bo Qiu agree to this retraction. Xi-Wen Zhang has not responded to any correspondence from the Editor about this retraction.

